# Porcine reproductive and respiratory syndrome virus genetic variability a management and diagnostic dilemma

**DOI:** 10.1186/s12985-021-01675-0

**Published:** 2021-10-18

**Authors:** Jessica Risser, Matthew Ackerman, Robert Evelsizer, Stephen Wu, Byungjoon Kwon, James Mark Hammer

**Affiliations:** 1 Elanco, Greenfield, IN USA; 2 Pork Vet Solutions, New Palestine, IN USA

**Keywords:** PRRSV, Genetic variability, Quasispecies, Darwinian theory, Recombination, Management practices

## Abstract

As genetic analysis becomes less expensive, more comprehensive diagnostics such as whole genome sequencing (WGS) will become available to the veterinary practitioner. The WGS elucidates more about porcine reproductive and respiratory syndrome virus (PRRSV) beyond the traditional analysis of open reading frame (ORF) 5 Sanger sequencing. The veterinary practitioner will require a more complete understanding of the mechanics and consequences of PRRSV genetic variability to interpret the WGS results. More recently, PRRSV recombination events have been described in the literature. The objective of this review is to provide a comprehensive outlook for swine practitioners that PRRSV mutates and recombines naturally causing genetic variability, review the diagnostic cadence when suspecting recombination has occurred, and present theory on how, why, and where industry accepted management practices may influence recombination. As practitioners, it is imperative to remember that PRRS viral recombination is occurring continuously in swine populations. Finding a recombinant by diagnostic analysis does not ultimately declare its significance. The error prone replication, mutation, and recombination of PRRSV means exact clones may exist; but a quasispecies swarm of variable strains also exist adding to the genetic diversity. PRRSV nonstructural proteins (nsps) are translated from ORF1a and ORF1b. The arterivirus nsps modulate the hosts’ immune response and are involved in viral pathogenesis. The strains that contribute the PRRSV replicase and transcription complex is driving replication and possibly recombination in the quasispecies swarm. Furthermore, mutations favoring the virus to evade the immune system may result in the emergence of a more fit virus. More fit viruses tend to become the dominant strains in the quasispecies swarm. In theory, the swine management practices that may exacerbate or mitigate recombination include immunization strategies, swine movements, regional swine density, and topography. Controlling PRRSV equates to managing the quasispecies swarm and its interaction with the host. Further research is warranted on the frequency of recombination and the genome characteristics impacting the recombination rate. With a well-defined understanding of these characteristics, the clinical implications from recombination can be detected and potentially reduced; thus, minimizing recombination and perhaps the emergence of epidemic strains.

## Introduction

Since being identified in the late 1980s, porcine reproductive and respiratory syndrome (PRRS) continues to represent a significant cost to the swine industry [1]. In the last 20 years, management of PRRS in the field has been focused on improving prevention of lateral PRRS infections and managing the PRRS immune response across the swine population. Use of filtration, herd closures, vaccination strategies, live virus exposure, and production of PRRS virus (PRRSV) negative breeding stock have been implemented to minimize PRRS impact on swine farms. Even with these measures, PRRSV still circulates at international, national, regional, and herd levels.

Cost effective technologies to fully analyze the PRRSV genome continue to be developed [2–9] emphasizing PRRSV’s genetic variability as a perplexing problem in PRRS disease management. Many classification and categorization schemes have been utilized to understand this genetic diversity [3, 4]. These classification schemes will most likely evolve with an increase implementation of whole genome sequencing (WGS). As WGS becomes less expensive, it will become more common place in swine veterinary practice. While WGS elucidates more about PRRSV than the traditional Sanger sequence of open reading frame (ORF) 5, the veterinary practitioner will require a more complete understanding of the mechanics and consequences of PRRSV genetic variability to fully interpret the WGS results.

PRRSV, as with many other ribonucleic acid (RNA) viruses, is prone to mutations and recombination. Point mutations occur frequently with each genomic replication. Nonstructural proteins (nsps) are generated from ORF1a and ORF1b. ORF1 comprises 75% of the PRRSV genome and codes for the PRRSV replicase and transcription complex (RTC) [10, 11]. It is also becoming clear that the nsps modulate the hosts’ immune response and are involved in viral pathogenesis [10, 11], as well as influence the mutation rate and propensity to recombine. In a recombination event, the strains contributing the nsps may be driving replication and perhaps recombination in the quasispecies swarm [10].

The objective of this review is to elucidate for the swine practitioners the current knowledge about PRRS natural genetic variability through point mutations and recombination. This review also covers the diagnostic tools necessary for a practitioner to ascertain if a recombination has occurred, and presents theory on how, why, and where industry accepted management practices may influence recombination and emergence of epidemic strains.

## Mechanisms of mutation and recombination

### PRRSV genome, viral structure, and function

PRRSV is a positive stranded RNA virus approximately 15 kilobase (kb) in length [12]. There are 11 ORFs that includes: the replicase complex (ORF1) and ORFs that code for structural proteins consisting of nucleocapsid (N, ORF 7), major structural proteins (GP5, ORF 5; M, ORF 6), minor structural proteins (GP2a, ORF 2a; E, ORF 2b; GP3, ORF3; GP4, ORF 4), and other minor protein ORF5a [12–14].

### PRRSV nonstructural proteins

**Fig. 1:**
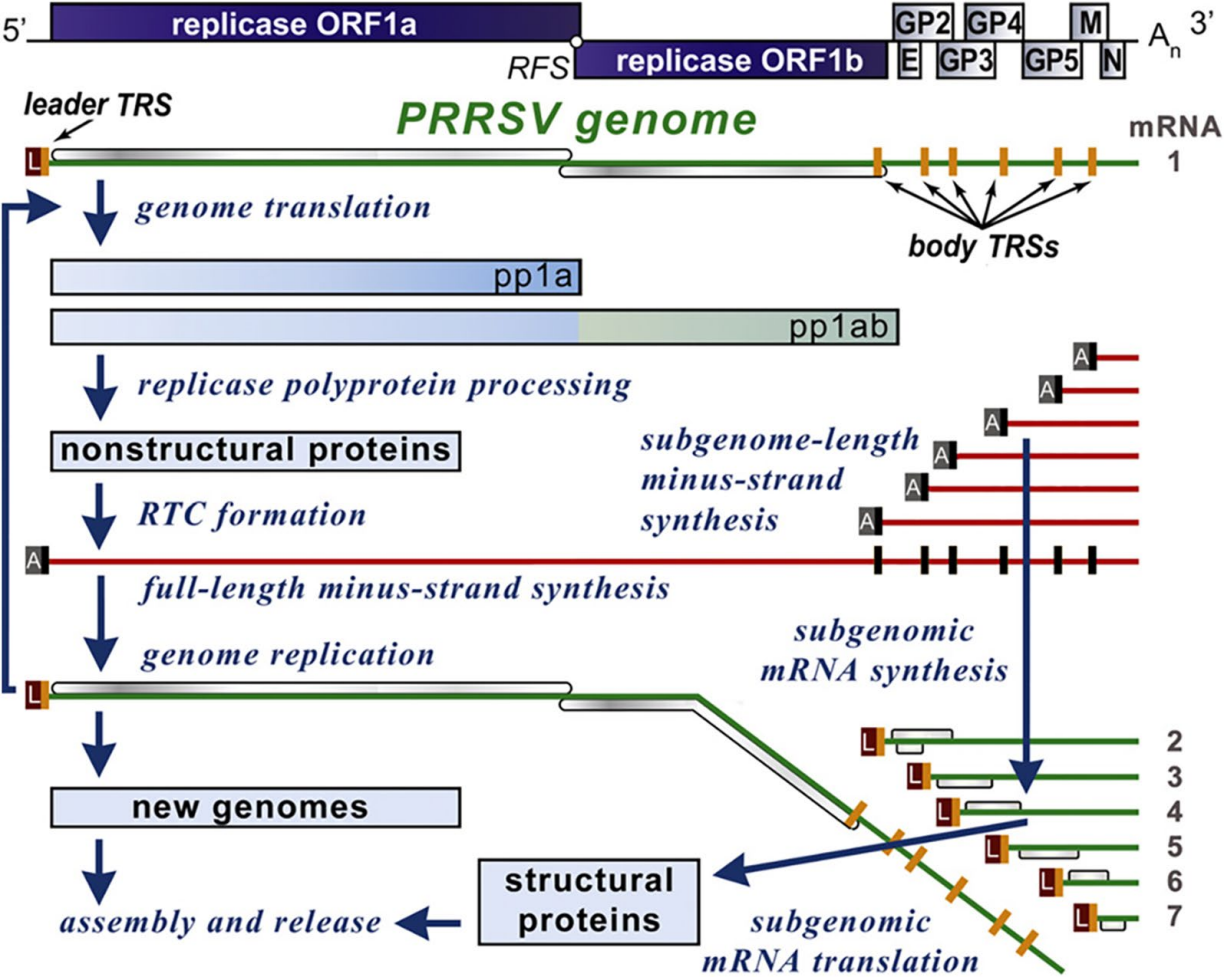
PRRS genome and replication cycle. The genomic replication cycle commences with the translation of polyproteins PP1a and PP1ab. These polyproteins are cleaved into the 14 nsps, which form the replication and transcription complex (RTC). The RTC transcribes the RNA minus strand and subgenomic mRNA (sgRNA). Reproduced with permission from Ref. [11]

The PRRSV genome and replication cycle is depicted in Fig. 1 [11]. ORF1 codes the PRRSV nonstructural replicase and transcription complex (RTC) [11, 15]. The genomic replication cycle commences with the translation of polyproteins, PP1a and PP1ab. These polyproteins are cleaved into the 14 nsps, which form the RTC. The RTC transcribes the RNA minus strand and subgenomic mRNA (sgRNA). The RTC switches to a discontinuous transcription, which allows the leader TRS (transcription regulatory sequence) to interact with the body TRS for subgenomic RNA synthesis [11]. This may favor recombination and variation in the structural proteins that are translated from the sgRNA [11]. These structural proteins are then assembled into the new viral particle.

### Inherent genetic variability

PRRSV replication demonstrates three key features facilitating genetic variability. These are rearrangement of host plasma cell membranes to establish viral replication complexes, synthesis and expression of genomic RNA, and transcription of sgRNA to efficiently express structural proteins [16, 17]. Viral genetic diversity is determined through the study of multiple viruses and host-dependent processes [18]. RNA viruses that allow for persistent infections may have a higher rate of recombination because a single host has an increased chance of acquiring multiple strains [19]. PRRS has a prolonged persistence phase where the virus can exist up to 250 days [15, 20–22].

## Estimation of the frequency of recombination

The two major types of mutation identified are: point mutations and complex genomic rearrangements (recombination) [23–25]. Preliminary reports demonstrate a 0.5% mutation change in ORF2-7 sequences in the short time between processing (birth) and pre-weaning (17–19 days of age) [26]. More recent WGS of field clinical samples indicates 4.55% prevalence of PRRS strain coinfections [27]. The study also found possible recombination in 6.5% of the 92 WGS successfully sequenced [27]. PRRSV specifically has a mutation rate between 4.71 × 10^2^ to 9.8 × 10^2^/synonymous sites/year [16, 28–31], which is about one mutation per replication cycle [32–35]. Rearrangement (recombination) is a mutation that replaces RNA sequences into a genome. RNA-dependent RNA polymerase (RdRp), encoded by PRRSV nsp9, is the PRRS replication polymerase [14, 23]. The RdRp is part of the PP1ab protein complex that makes up the RTC [14]. RNA recombination is facilitated by the RdRp as it switches from one RNA molecule to another during replication, particularly of the structural subgenomic mRNA (Fig. 1) [11, 19]. This is referred to as template switching and occurs at regions of high sequence similarity [19]. Therefore, template switching may occur more frequently when PRRS genomes with areas of high similarity exist in the quasispecies [19]. Template switching is a natural process of RNA viruses [36].

## Tools available to identify recombination events

Researchers [37–43] have reported recombination from the field through the application of multiple molecular evaluations. Veterinary diagnostic laboratories in the United States have recently reported cases demonstrating the benefits of WGS to define recombination of attenuated vaccine strain and wild-type (wt) strain or different attenuated vaccine strain [44, 45]. These case reports begin with a discrepancy between the clinical observations and expected results of the respective immunization programs, thus prompting further diagnostic investigations. A number of analytical and statistical tools are needed to distinguish between recombination and accumulation of point mutations [46, 47].

Discrepancies between the routine monitoring or surveillance and clinical observations is evidence of a potential immune escape or recombination event. In herds previously wt exposed or attenuated live vaccinated, a root cause of PRRS requires histopathological interstitial pneumonia and/or immunohistochemical evidence of PRRS virus in the lung tissue. In cases of potential recombination, it is likely to find multiple strains [19]. These clinical samples may include new strains, previous identified strains, strains with point mutations, or potential recombined strains. Multiple samples or laboratory submissions from a herd may be needed to enumerate strains involved. The dominant strain may change over time through point mutations or recombination while less prevalent strains, once undetected, may be implicated.

**Fig. 2:**
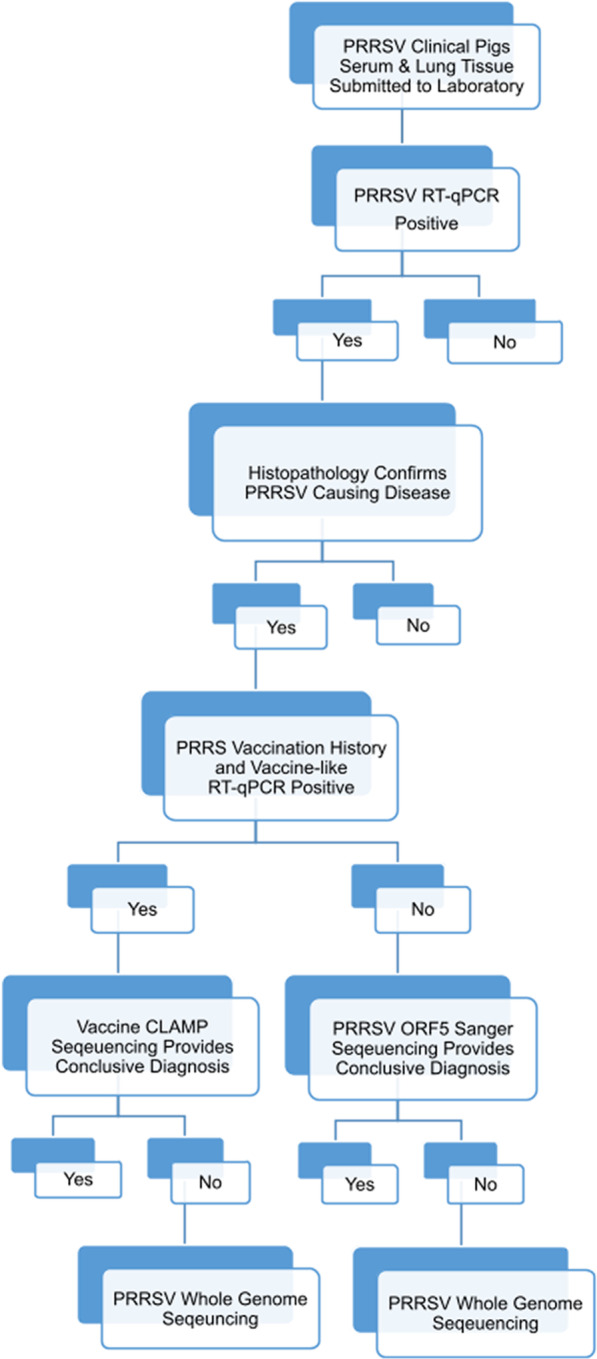
The diagnostic steps to investigate abhorrent clinical findings in monitoring and surveillance samples. Figure created by author

The steps to investigate abhorrent clinical findings in monitoring and surveillance samples is depicted in Fig. 2. A quantitative reverse transcription polymerase chain reaction (RT-qPCR) verifies the presence of PRRSV. Depending on the case’s vaccine history, vaccine-like preferential RT-qPCR may be performed. The presence of wt virus is deduced through comparison of the cycle threshold (ct) values of samples versus the vaccine-like preferential RT-qPCR. An approximate difference of 6 ct values between the RT-qPCR and vaccine-like preferential RT-qPCR suggest multiple viruses are present in the sample, although these approximations may vary between vaccines and techniques [48]. Sanger sequencing [49] or a vaccine-CLAMP [50] (to block vaccine virus) of the ORF5 region is completed to determine which strain is involved in the outbreak, potential number of strains, and the relatedness to previous strains in the herd through phylogenetic classification (dendrogram). The dendrogram helps determine if a common ancestor exists, and how long it may have existed [4]. Classification of PRRSV using the ORF5 sequencing and restriction fragment length polymorphism (RFLP) pattern is well recognized but has limitations. An ORF5 sequence is not sufficient to identify a recombination event; because of ORF5’s hypervariability, the small percent of the genome analyzed, and the limited analysis of any structural and functional changes [3, 4].

If the initial diagnostics are inconclusive in determining root cause of clinical observations, WGS is utilized for a more comprehensive analysis. Multiple software programs (i.e. ClustalX, SimPlot, Bootscan) are utilized to evaluate suspected recombinant and previously recognized wt strains from the population [41, 42, 44, 51, 52]. Alternatively, various novel sequencing technologies have been developed and continue to be refined to characterize large sequences, be of low cost per base, and have short turnaround time [9]. Researchers have described the development of WGS for clinical samples containing multiple PRRSV strains [9]. The WGS results are compared to the known attenuated vaccine strains, and the herd’s previously identified wt strains. Precautions are needed in WGS evaluations as analysis may be affected by factors such as the ct value of the sample (recommended < 25), sample type (preference serum, then lung, oral fluids, and lastly processing fluids), overall sample quality, and PRRSV RNA integrity [9].

**Fig. 3:**
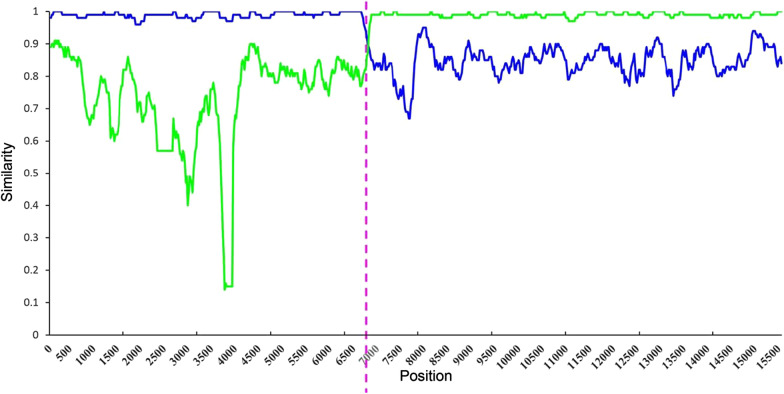
Similarity plot analysis using IA70388-R (recombinant) strain of PRRSV as the query sequence against IA76950-WT (wt, blue line) strain and an attenuated live vaccine-like strain (green line). A recombination breakpoint is shown with a purple dotted line and the location is underscored at the nucleotide site. Reproduced from Ref. [44]

Figure 3 represents an illustration of an analysis of a recombinant PRRSV. The similarities between a recombinant (IA70388-R) to the previous wt (IA76950-WT) and an attenuated live vaccine-like strain [44] are illustrated. The breakpoint (represented by the purple line in Fig. 3.) is where sequence similarity crossed [44]. The strain indicated by the top line represents the recombinant strain [51] and is the sequence’s best approximation. The “major parent” strain contributes the majority of its genomic sequence to the recombinant strain [53]. The “minor parent” strain contributes a smaller portion of its genomic sequence to the recombinant strain [53]. In Fig. 3, the major parent is IA76950-WT, while the attenuated live vaccine-like strain is the minor parent.

As WGS becomes more common, more PRRSV recombination may be identified but the clinical significance of the recombination will have to be discerned. Clinical significance should be elucidated through a well performed clinical challenge study of strains identified (recombinant strain, major and minor parent strains). The challenge study should include defined clinical outcomes, lung pathology, and virologic and serological evaluations [42]. However, a challenge study is very difficult and expensive to perform. A challenge study should be pursued if significant losses continue to occur over a large region for an extended time.

## Possible consequences of mutation (Emergence of a dominant strain)

Two similar but fundamentally different theories have been put forth to explain the phenomena of strain emergence. These theories are survival of the fittest, the Darwinian Theory, and the Quasispecies Theory based on maintaining equilibrium in the quasispecies swarm. Researchers debate about which theory drives the emergence (fitness) of a new PRRSV strain. It is likely a combination of these theories, and both quasispecies and host factors at work to allow emergence of a more fit strain.

### Quasispecies

RNA viruses exist as quasispecies swarm, which is a group of similar but not identical virus particles [23]. When dissimilar viruses infect a herd, the virus with greater fitness will supersede other less fit viruses [32]. Quasispecies exist because of the inherent characteristics of mutation rate and viral recombination [5, 54–56]. Quasispecies are natural and normal in a PRRSV infection. The goal, when managing PRRS in swine herds, should be to have a similar quasispecies not a dissimilar one. The more dissimilar the quasispecies, higher the likelihood of a dually infected animal which increases the risk of recombination [19].

The quasispecies theory exposes a state of equilibrium where a majority strain exists in the quasispecies swarm [57]. A majority strain’s survival is a function of mutation rate, frequency of mutation at a specific site, and the fitness the genetic diversity imparts. The quasispecies is influenced by internal (viral) factors and external (host) factors. The internal factors present a phenotypic expression which then interacts with the external factors producing the most fit strain under the selection pressures [57]. Internal factors favoring more rapid replication, replication accuracy, or immune escape may favor strains to become dominant and therefore emerge. The external environment is primarily the host’s immune response toward the quasispecies that influences the emergence of strains.

### Darwinian theory

When applying the Darwinian Theory [57], the quasispecies swarm is pressured to escape the host’s immune response to produce a more “fit” virus [5, 57–59]. The pig’s immune response thus may drive antigenic diversity through Darwinian selection pressure [2, 40, 59]. As the host’s immune system attempts to eliminate infection and PRRSV attempts to replicate in the body, a mutation favoring less immune pressure may allow emergence of a more “fit” virus thus increasing their numbers in the quasispecies swarm. This increase forces the quasispecies equilibrium toward a mutant less immunologically recognized favoring the “escape mutant” strain in the quasispecies swarm [57]. Other advantageous mutations may result in a more efficient replicase, thus being able to outnumber other isolates favoring its numbers in the swarm’s equilibrium. Because these conditions exist, new strains are expected to emerge [5].

As alluded to previously, herd immunity and genetic mutation/recombination can explain strain emergence. Herd immunity although important to control PRRSV may drive emergence of a more “fit” strain as the virus survives in the host tissues for extended periods. Genetic mutation and recombination are also implicated in the emergence of new PRRSV strains [5, 16, 30, 40, 60]. As molecular techniques improve and become less costly, the ability to detect variants may increase. Further research will need to determine if PRRSV strains with inherently greater capacity for genetic variability are within some quasispecies swarm but not currently detectable. Elucidation of genomic regions conferring these qualities is yet to be fully described.

## Cases study

A diagnostic case presented with a history of discrepancies in clinical observation, practitioner’s expectations, and surveillance diagnostic results. Sequences of three samples revealed a range of high similarity to an attenuated live vaccine strain previously used in the herd. Table 1 demonstrates the RT-qPCR and ORF5 sequence similarity data resulting from the three samples. Only two of the three samples were positive for the vaccine-like preferential RT-qPCR.

**Table 1:** Percent similarity of three submissions over time from the same herd

Samples	RT-qPCR	Sanger sequencing ORF5 (% similarity)	Vaccine-like preferential RT-qPCR	Difference in ct value of preferential RT-qPCR and RT-qPCR
1	27.8	94.9	+ (ct 33.4)	5.6
2	26.7	97.3	−	N/A
3	22.7	97.7	+ (ct 26.9)	4.2

**Fig. 4:**
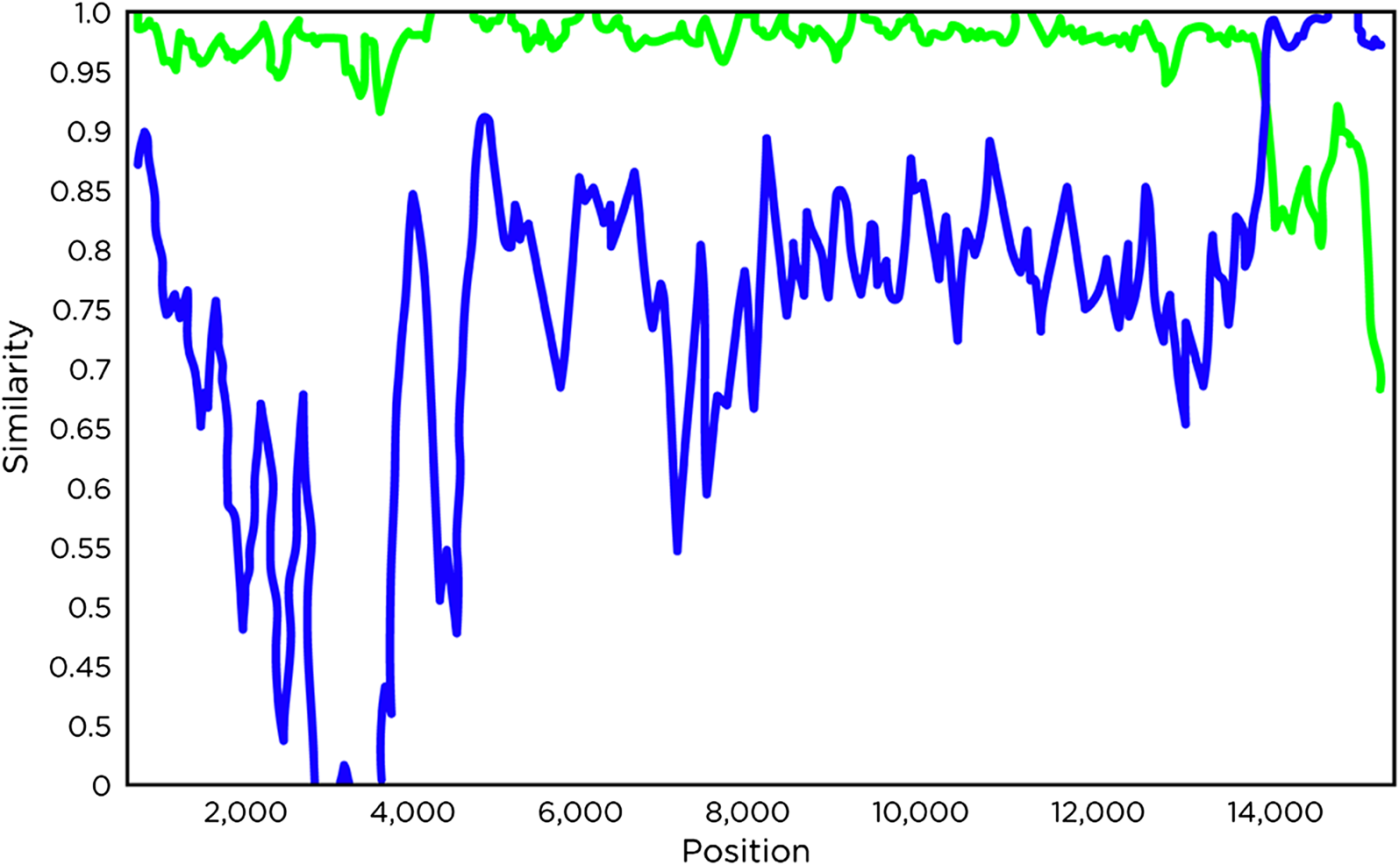
Similarity plot analysis using the strain from sample number 2 (recombinant) as the query sequence against the last know wild-type (lwt) strain (green line) and the previously used attenuated live vaccine-like strain (blue line). Figure created by author

The sequence similarity and ct value differences suggested the presence of three strains: a vaccine-like (sample #3), a wt (sample #1), and a recombinant (sample #2). WGS was pursued to further elaborate these findings. Sample number 2 was chosen for WGS because it had a high percent similarity on ORF5 sequence and a negative vaccine-like preferential RT-qPCR. The same cadence of diagnostics was conducted on the temporally last known wt (lwt) break isolate. Figure 4 demonstrates the similarity of the sample number 2 strain to lwt and attenuated live vaccine-like strain. The analysis identified the lwt strain as the major parent, and the attenuated live vaccine-like strain as the minor parent. The analysis identified a recombinant, but clinical relevance is still undetermined for the case.

A case example from the literature demonstrates recombinant strain evaluations for clinical significance. The epidemiological evaluation of sequences in Shandong Province China from 2014–2015 revealed three recombinants [61]. WGS and animal studies were conducted to compare the three recombinants to three known strains [61]. The variation in clinical outcome of the three recombinant strains may have resulted from different recombination breakpoints, which changed the major parent strain contributing the ORF1 (bp between 10,000 and 12,000) region [61]. The ORF1 region originated from the major parent strain while the remainder was contributed by the minor parent [61].

In this instance the pathogenicity of the recombinant was driven by the major parent strain contributing ORF1 region. ORF1 determines the nsps which drives replication and best predicted the recombinant strain’s clinical outcome [15]. Theoretically, the parent strain that contributes the replication complex, which is driving replication, influences the presence in the quasispecies swarm and the interaction with the immune system [10].

## Proposed practices that influence recombination

Controlling PRRS requires one to manage the quasispecies swarm and its interaction with the host. From this perspective one can postulate managing the immunity in the swine herd may influence the quasispecies [46]. Practices that may exacerbate or mitigate recombination could include: the timing of vaccine administration (breeding herd, grow-finish, pre-weaning, or post-weaning), live virus inoculation verses attenuated vaccination, full or partial vaccine dosing, selection of vaccine, frequency of vaccine administration, frequency of animal entry, and other wt introductions. Ideally the least pathogenic strain, the strain with the least negative consequences to production, can maintain dominance in the quasispecies swarm while continuously stimulating the host immune system.

### Immunization strategies

Breeding herd vaccination strategy starts with managing the immunity in the gilt production herd. Within the last several years, the industry has succeeded at the production of PRRSV negative breeding stock. However, the naïve gilts undergo various immunization strategies before entry into PRRSV positive herds. Gilt acclimatization is a critical time point for building PRRSV immunity of the breeding herd. This strategy is to minimize circulation of endemic strain(s) within the herd. These immunization strategies are variable between veterinary and production systems. Without a convenient antibody test to assess neutralizing immunity, success is judged through quiescence of clinical signs, maintenance of sow herd performance, and/or RT-qPCR negative piglet populations at birth. However, these assessments do not always equate to population immunity. This perceived lack of population immunity has led practitioners to adapt various vaccination schemes; some include wt virus inoculation and/or a combination of multiple attenuated live vaccines. The consequences or rewards are poorly documented in the literature.

The US swine industry has moved from production cycle vaccinations to whole herd calendar vaccinations becoming the standard for maintaining PRRS control with variable success. The different philosophy of these vaccination schemes needs to be considered in managing the immune status of the entire herd (farrow to finish). Initial consideration should be on the risk of lateral introductions, and success of biosecurity measures in the various stages of production. Breeding herds are successful, as measured by negative RT-qPCR results on pigs at weaning, at achieving clinical protection with whole herd calendar sow vaccination, gilt acclimatization, and biosecurity measures [62, 63]. The challenge in these herds is the potential waxing and waning maternal antibody levels; that may influence post-weaning PRRS expression and secondary bacterial clinical control. If vaccination on a production cycle, 6 weeks prior to farrowing, is included in the breeding herd program; more consistent maternal antibody levels may be achieved subsequentially resulting in a more homogenous immune status of the post-weaning population. Most post-weaning pigs located in high-risk PRRS geographical areas are attenuated live vaccinated prior to weaning; however, wt exposure may occur as early as the day of weaning. These pigs are relying on the maternal antibodies to bridge the gap between the time of vaccination and wt exposure [64]. Few reports are available to document the efficacy of these approaches [65].

The whole herd calendar vaccination program administers attenuated vaccines through quarterly mass vaccinations of breeding herds [66, 67]. However, over time and due to the lack of perceived attenuated vaccines’ heterologous protection, some have developed an attenuated vaccine rotation strategy [68, 69]. This approach utilizes different attenuated PRRSV strain 1, 2, 3, or 4 times a year. This strategy ignores the known risk for in utero transmission and PRRSV mutation and recombination [70]. Breeding herds have shown to be prone to point mutations in the ORF5 region, regardless of any amino acid changes, with transmission of PRRSV between the sow and litter of pigs in utero [70]. Pigs from bred sows vaccinated with PRRS attenuated live vaccine may become persistent up to 260 days [71]. Most challenge evaluations in pregnant sows are in naive or minimally vaccinated sows and do not typically assess piglet post-weaning impact of vaccination during pregnancy. Furthermore, the literature has shown that utilization of multiple attenuated vaccines in the same population at the same time can increase the possibility of dually infected pigs, in utero transmission, and exacerbate a viral recombination environment [70, 72, 73]. These concepts support that the grow-finish pigs maintain the same vaccine as the breeding herd. This is likely to minimize live and attenuated strains present and continue supporting a dominant strain in the quasispecies swarm.

Based on the quasispecies theory, a more similar quasispecies is more desirable over a dissimilar one. The administration of attenuated live vaccination during a wt infection is a practice to mitigate a clinical outbreak. There are studies on the value of vaccination to decrease duration of shedding wt virus in growing pigs [74, 75]. The risk of recombination in this situation is not documented in literature. Additionally, further research is needed to explain the risk of recombination in breeding herds when an attenuated vaccine is administered during a wt infection. This risk is most likely variable based on the parity, prior immune status, and trimester of gestation of the breeding animal [70, 71, 76, 77].

In grow-finish pigs, use of multiple attenuated vaccines may be less of a contributing factor to recombination, especially in three site production systems [78]. Factors, that may increase risk of recombination, to consider before implementation of a new attenuated vaccine in grow-finish include: the breeding herd PRRS status, the timing of vaccination post-weaning versus pre-weaning, the population size, and other attenuated vaccines administered to the same swine population. Conversely, attributes of grow-finish pigs that may reduce the risk of recombination include that those populations have a shorter life span, are raised in all-in-all-out facilities, and have unidirectional pig flow to market. All are mitigating or exacerbating factors that implicate the growing herd in recombination [79].

### Reduced dose of vaccines

Prior to a new vaccine reaching swine barns, vaccine manufacturers analyze the new product on various parameters. Dose titration studies are performed to determine the most efficacious antigen level in an individual dose. Briefly, pigs are vaccinated with experimental vaccines of various antigen levels. After challenge, the pigs are assessed on the clinical outcome, immune response, and disease lesions to determine the appropriate antigen level. The antigenic mass in the attenuated vaccine is determined to stimulate an efficacious immune response through classical challenge studies. The administration of lower antigenic mass may not produce an efficacious immune response.

Partial dosing of attenuated PRRS vaccines has become widespread in US swine production despite significantly greater average daily gain, numerical better nursery mortality and feed conversion when a full dose is administered [80]. The risk of partial dosing on the safety and efficacy of a vaccine is unknown; in addition, the impact the partial dose has on the quasispecies and recombination has not been documented in the literature.

The maintenance of an attenuated dominant strain in the quasispecies and a neutralizing immune response should be the goal of a PRRS management program. If a population does not reach an efficacious immune response, persistence in lymphoid tissues may be variable and extended. This could lead to pathogenic strain dominance in the quasispecies, a highly variable mixed infection, and recombination. The higher the likelihood of a single host infected with multiple strains, the higher the likelihood of recombination [19, 27]. A proven immunogenic dose should be used to maintain the attenuated PRRSV vaccine within a herd. Further investigations are needed to determine the impact of partial dosing on recombination.

### Swine movements

An additional aspect of controlling the quasispecies and minimizing mutations/recombination is the consideration of animal movements, and swine density. These movements start with the frequency of replacement gilt entry and continue through the flow to market. If we consider the prior health status and acclimatization of the replacement gilts, the risk is that new naïve or less than immune gilts entering in a breeding herd cause fluctuation in quasispecies [79]. This begins the cascade of disease through the breeding pyramid as exposed pigs move between farms of different production phases through the commercial growing herd, which may consist of a nursery and finishing system or wean to finish sites [79]. The risk of recombination also applies to grow-finish flows in which multiple sources of pigs are comingled within the same site. Depending on their PRRS status, this practice brings together populations with different PRRSV strains and varying immunity statuses. This common production practice may impact the dissimilarity of the quasispecies, and subsequently impact clinical observations and performance outcomes. Under conditions where PRRSV is clinically controlled in grow-finish herds, virus may still be present in some infected pigs as a persistent state in the lymphoid tissues. Clinical outcome may depend on the virulence of the strain causing infection and on management practices. Thus, it is possible that PRRSV is circulating without clear clinical signs. This circulation, in combination with attenuated live vaccination practices and lateral entries of new wt viruses, can potentiate recombination events.

### Lateral transmission

Transmission of PRRSV within swine dense areas is a common occurrence [79]. When susceptible pigs are moved to a farm with any combination of the cited risk factors the opportunity to spread disease and allow new PRRSV strains to emerge is being documented [24, 79, 81]. When PRRSV spreads to another farm in a region with low or incomplete immunity, the introduced strain may create a clinical outbreak enhancing potential mutation or recombination as the virus infects immunologically naive pigs. Because the immune pressure has changed the introduced strain may survive, thrive, and emerge as a dominant strain continuing to cause clinical outbreaks in the herd or region. Others have also observed regional disease transmission following pig movements [81]. Although, this would depend on the region’s probability for aerosol transmission of wt or vaccine-like PRRSV. Factors contributing to region spread are the diversity of production types (breeding vs. grow-finish), variation of PRRS control mechanisms, the swine density, and geography. The literature documents the distance between farms and natural barriers impacts PRRSV spread [79, 81–83]. Topography, vegetation, pig density, and proximity to roads are factors contributing to the spread of disease [79, 81–83]. In the production systems where ORF5 sequences were evaluated, 1.3% of the sequences were recombinants with almost half originating from one production system and 82% originating from the breeding to wean (sow) units [79]. Furthermore, many recombinant sequences were in the geographic area from sow units. Thus, control of PRRSV at the sow unit should reduce area infection. Review of large data sets appears to point to a lack of sow herd stability allowing PRRSV to circulate in a geographic region. Any practice that minimizes the transmission of PRRSV will also minimize the quasispecies dissimilarity and presumably minimize recombination. These are important considerations in minimizing disease transmission within a herd and regionally.

## Conclusion

PRRSV replication lends itself to genetic variability through point mutations, template switching, immune escape, and dissimilar quasispecies. As attenuated vaccine strains are derived from wt viruses, it comes as no surprise that various wt and attenuated strains have been reported to recombine in vitro and on farms worldwide [40, 41, 43, 44, 68, 72].

The objective of this review was to inform swine veterinarians with the knowledge that PRRS will mutate and recombine naturally causing genetic variability, review the diagnostic cadence necessary when recombination is suspected, and present theory on how, why, and where industry accepted management practices may influence recombination which may cause a change in the dominant variant in the viral quasispecies. PRRSV potential to mutate and recombine is normal. The industry needs to recognize some accepted practices may affect PRRSV transmission and recombination potential. When considering to minimize mutations, managing the quasispecies and its interaction with the host is a paramount. The industry’s focus on biosecurity and control/elimination programs is necessary but has proved insufficient to fully manage PRRS as evident by the continued evolution of PRRSV [6, 32].

Aspects of managing the quasispecies swarm should be minimizing multiple strains in a herd, maintaining an attenuated or low pathogenic strain, and maintaining a neutralizing immune response as a goal of any successful PRRS management program. Thus, a proven immunogenic dose should be used to maintain a similar quasispecies in a herd, if not a region depending on the swine density and geography. Immunization strategy, swine movements, regional swine density and topography all need to be considered as factors in managing PRRSV. It is time to use new resources to think not just of the pig population, but of the viral population. More open sharing of data can elucidate insight into the management of PRRSV in swine herds, as well as, managing the quasispecies swarm to decrease the potential for the emergence of PRRSV strains.

As practitioners, it is imperative to remember that viral recombination is occurring continuously in swine herds. Discrepancies between diagnosis and clinical observations are the first evidence of a potential recombination event requiring further examination. If PRRS is substantiated through histopathology, a series of diagnostic tools are employed to link the strains in the diagnostic samples to previous known herd strain(s). Clinical relevance of genetic changes found in diagnostic investigations require a proper genetic evaluation. Multiple diagnostic tests and statistical analysis are needed to differentiate a recombination from a series of point mutations. The rate of recombination, the genome characteristics impacting the recombination rate, the clinical implications from recombination, and how to minimize recombination are the quandary for the scientific community to clarify and the veterinary profession to implement.

## Data Availability

Data sharing is not applicable to this article as no datasets were generated or analyzed during the current study.

## Abbreviations

ct: Cycle time
lwt: Last known wild-type
nsps: Nonstructural proteins
ORF: Open reading frame
PRRS: Porcine reproductive and respiratory syndrome
PRRSV: Porcine reproductive and respiratory syndrome virus
RdRp: RNA-dependent RNA polymerase
RFLP: Restriction fragment length-polymorphism
RNA: Ribonucleic acid
RT-qPCR: Quantitative reverse transcription polymerase chain reaction
RTC: Replication and transcription complex
sgRNA: Subgenomic RNA
TRS: Transcription regulatory sequence
WGS: Whole genome sequencing
wt: Wild-type

## References

[1] Holtkamp D, Kliebenstein J, Neumann E, Zimmerman J, Rotto H, Yoder T (2013). Assessment of the economic impact of porcine reproductive and respiratory syndrome virus on United States pork producers. J Swine Health Prod.

[2] Kapur V, Elam MR, Pawlovich TM, Murtaugh MP (1996). Genetic variation in porcine reproductive and respiratory syndrome virus isolates in the midwestern United States. J Gen Virol.

[3] Wesley RD, Mengeling WL, Lager KM, Clouser DF, Landgraf JG, Frey ML (1998). Differentiation of a porcine reproductive and respiratory syndrome virus vaccine strain from North American field strains by restriction fragment length polymorphism analysis of ORF 5. J Vet Diagn Investig.

[4] Cha SH, Chang CC, Yoon KJ (2004). Instability of the restriction fragment length polymorphism pattern of open reading frame 5 of porcine reproductive and respiratory syndrome virus during sequential pig-to-pig passages. J Clin Microbiol.

[5] Paploski IAD, Corzo C, Rovira A, Murtaugh MP, Sanhueza JM, Vilalta C (2019). Temporal dynamics of co-circulating lineages of porcine reproductive and respiratory syndrome virus. Front Microbiol.

[6] Shi M, Lam TT-Y, Hon C-C, Hui RK-H, Faaberg KS, Wennblom T (2010). Molecular epidemiology of PRRSV: a phylogenetic perspective. Virus Res.

[7] Shi M, Lam TT-Y, Hon C-C, Murtaugh MP, Davies PR, Hui RK-H (2010). Phylogeny-based evolutionary, demographical, and geographical dissection of North American type 2 porcine reproductive and respiratory syndrome viruses. J Virol.

[8] Kinsley K, Guggenbiller D, Weiss D, Nimmo R, Kim BK, editors. Reviewing a novel approach to classifying porcine reproductive and respiratory syndrome virus—MJPRRS Grouping Technology. Annual Meeting of American Association Swine Veterinarians: Standing on the Shoulders of Giants: Collaboration and Teamwork; 2016.

[9] Zhang J, Zheng Y, Xia XQ, Chen Q, Bade SA, Yoon KJ (2017). High-throughput whole genome sequencing of porcine reproductive and respiratory syndrome virus from cell culture materials and clinical specimens using next-generation sequencing technology. J Vet Diagn Invest.

[10] An T-Q, Li J-N, Su C-M, Yoo D (2020). Molecular and cellular mechanisms for PRRSV pathogenesis and host response to infection. Virus Res.

[11] Fang Y, Snijder EJ (2010). The PRRSV replicase: exploring the multifunctionality of an intriguing set of nonstructural proteins. Virus Res.

[12] Montaner-Tarbes S, Del Portillo HA, Montoya M, Fraile L (2019). Key gaps in the knowledge of the porcine respiratory reproductive syndrome virus (PRRSV). Front Vet Sci.

[13] Lunney JK, Fang Y, Ladinig A, Chen N, Li Y, Rowland B (2016). Porcine reproductive and respiratory syndrome virus (PRRSV): pathogenesis and interaction with the immune system. Annu Rev Anim Biosci.

[14] Dokland T (2010). The structural biology of PRRSV. Virus Res.

[15] Yun SI, Lee YM (2013). Overview: replication of porcine reproductive and respiratory syndrome virus. J Microbiol.

[16] Kappes MA, Faaberg KS (2015). PRRSV structure, replication and recombination: origin of phenotype and genotype diversity. Virology.

[17] Yuan S, Murtaugh MP, Faaberg KS (2000). Heteroclite subgenomic RNAs are produced in porcine reproductive and respiratory syndrome virus infection. Virology.

[18] Sanjuán R, Domingo-Calap P (2016). Mechanisms of viral mutation. Cell Mol Life Sci.

[19] Simon-Loriere E, Holmes EC (2011). Why do RNA viruses recombine?. Nat Rev Microbiol.

[20] Allende R, Laegreid WW, Kutish GF, Galeota JA, Wills RW, Osorio FA (2000). Porcine reproductive and respiratory syndrome virus: description of persistence in individual pigs upon experimental infection. J Virol.

[21] Batista L, Pijoan C, Dee S, Olin M, Molitor T, Joo HS (2004). Virological and immunological responses to porcine reproductive and respiratory syndrome virus in a large population of gilts. Can J Vet Res.

[22] Horter DC, Pogranichniy RM, Chang CC, Evans RB, Yoon KJ, Zimmerman JJ (2002). Characterization of the carrier state in porcine reproductive and respiratory syndrome virus infection. Vet Microbiol.

[23] Figlerowicz M, Alejska M, Kurzyńska-Kokorniak A, Figlerowicz M (2003). Genetic variability: the key problem in the prevention and therapy of RNA-based virus infections. Med Res Rev.

[24] Paploski I, Corzo C, Rovira A, Murtaugh M, Sanhueza J, Smith E, et al., editors. Making epidemiological sense out of large datasets of PRRS seqeunces. In: Allen D Leman Swine conference. Saint Paul: University of Minnesota; 2018.

[25] Nagy PD, Simon AE (1997). New insights into the mechanisms of RNA recombination. Virology.

[26] Kikuti M, editor. Understanding PRRSv diversity at the pig and litter levels using whole-genome sequencing. In: Allen D Leman Swine conference proceedings; 2020 September 19–22. Saint Paul: The University of Minnesota College of Veterinary Medicine and University of Minnesota Extension; 2020.

[27] Lalonde C, Provost C, Gagnon CA (2020). Whole genome sequencing of porcine reproductive and respiratory syndrome virus (PRRSV) from field clinical samples improves the genomic surveillance of the virus. J Clin Microbiol.

[28] Forsberg R (2005). Divergence time of porcine reproductive and respiratory syndrome virus subtypes. Mol Biol Evol.

[29] Forsberg R, Oleksiewicz MB, Petersen A-MK, Hein J, Bøtner A, Storgaard T (2001). A molecular clock dates the common ancestor of European-type porcine reproductive and respiratory syndrome virus at more than 10 years before the emergence of disease. Virology.

[30] Forsberg R, Storgaard T, Nielsen HS, Oleksiewicz MB, Cordioli P, Sala G (2002). The genetic diversity of European type PRRSV is similar to that of the North American type but is geographically skewed within Europe. Virology.

[31] Hanada K, Suzuki Y, Nakane T, Hirose O, Gojobori T (2005). The origin and evolution of porcine reproductive and respiratory syndrome viruses. Mol Biol Evol.

[32] Brar M, Shi M, Murtaugh MP, Leung F (2015). Evolutionary diversification of type 2 porcine reproductive and respiratory syndrome virus. J Gen Virol.

[33] Duffy S, Shackelton LA, Holmes EC (2008). Rates of evolutionary change in viruses: patterns and determinants. Nat Rev Genet.

[34] Drake JW (1993). Rates of spontaneous mutation among RNA viruses. Proc Natl Acad Sci.

[35] Sanjuán R, Nebot MR, Chirico N, Mansky LM, Belshaw R (2010). Viral mutation rates. J Virol.

[36] Martín-Valls GE, Kvisgaard LK, Tello M, Darwich L, Cortey M, Burgara-Estrella AJ (2014). Analysis of ORF5 and full-length genome sequences of porcine reproductive and respiratory syndrome virus isolates of genotypes 1 and 2 retrieved worldwide provides evidence that recombination is a common phenomenon and may produce mosaic isolates. J Virol.

[37] Zhao H, Han Q, Zhang L, Zhang Z, Wu Y, Shen H (2017). Emergence of mosaic recombinant strains potentially associated with vaccine JXA1-R and predominant circulating strains of porcine reproductive and respiratory syndrome virus in different provinces of China. Virol J.

[38] Yuan S, Murtaugh MP, Schumann FA, Mickelson D, Faaberg KS (2004). Characterization of heteroclite subgenomic RNAs associated with PRRSV infection. Virus Res.

[39] Liu D, Zhou R, Zhang J, Zhou L, Jiang Q, Guo X (2011). Recombination analyses between two strains of porcine reproductive and respiratory syndrome virus in vivo. Virus Res.

[40] Yuan S, Nelsen CJ, Murtaugh MP, Schmitt BJ, Faaberg KS (1999). Recombination between North American strains of porcine reproductive and respiratory syndrome virus. Virus Res.

[41] Li B, Fang L, Xu Z, Liu S, Gao J, Jiang Y (2009). Recombination in vaccine and circulating strains of porcine reproductive and respiratory syndrome viruses. Emerg Infect Dis.

[42] Zhang Q, Bai J, Hou H, Song Z, Zhao Y, Jiang P (2017). A novel recombinant porcine reproductive and respiratory syndrome virus with significant variation in cell adaption and pathogenicity. Vet Microbiol.

[43] Bian T, Sun Y, Hao M, Zhou L, Ge X, Guo X (2017). A recombinant type 2 porcine reproductive and respiratory syndrome virus between NADC30-like and a MLV-like: genetic characterization and pathogenicity for piglets. Infect Genet Evol.

[44] Wang A, Chen Q, Wang L, Madson D, Harmon K, Gauger P (2019). Recombination between vaccine and field strains of porcine reproductive and respiratory syndrome virus. Emerg Infect Dis.

[45] Mor S, Rovira A. Whole genome sequencing provides answer in PRRSV investigation. National Hog Farmer: Informa Business Media; 2020. https://www.nationalhogfarmer.com/animal-health/whole-genome-sequencing-provides-answer-prrsv-investigation.

[46] Domingo E, Holland JJ (1997). RNA virus mutations and fitness for survival. Annu Rev Microbiol.

[47] Brar MSSM, Hui RKH, Leung FCC (2014). Genomic evolution of porcine reproductive and respiratory syndrome virus (PRRSV) isolates revealed by deep sequencing. PLoS ONE.

[48] Gauger P, Harmon K, editors. PRRS CLAMP: molecular diagnostic tools to distinguish wild-type and vaccine strains of PRRSV. 52nd Annual meeting of the American Association of Swine Veterinarians; 2021; Virtual.

[49] Sanger F, Nicklen S, Coulson AR (1977). DNA sequencing with chain-terminating inhibitors. Proc Natl Acad Sci U S A.

[50] Orum H (2000). PCR clamping. Curr Issues Mol Biol.

[51] Lole KS, Bollinger RC, Paranjape RS, Gadkari D, Kulkarni SS, Novak NG (1999). Full-length human immunodeficiency virus type 1 genomes from subtype C-infected seroconverters in India, with evidence of intersubtype recombination. J Virol.

[52] Han G, Xu H, Wang K, He F (2019). Emergence of two different recombinant PRRSV strains with low neutralizing antibody susceptibility in China. Sci Rep.

[53] Chen Y, Chen YF (2014). Extensive homologous recombination in classical swine fever virus: a re-evaluation of homologous recombination events in the strain AF407339. Saudi J Biol Sci.

[54] Chang CC, Yoon KJ, Zimmerman JJ, Harmon KM, Dixon PM, Dvorak CMT (2002). Evolution of porcine reproductive and respiratory syndrome virus during sequential passages in pigs. J Virol.

[55] Goldberg TL, Lowe JF, Milburn SM, Firkins LD (2003). Quasispecies variation of porcine reproductive and respiratory syndrome virus during natural infection. Virology.

[56] Lauring AS, Andino R (2010). Quasispecies theory and the behavior of RNA viruses. PLoS Pathog.

[57] Más A, López-Galíndez C, Cacho I, Gómez J, Martínez MA (2010). Unfinished stories on viral quasispecies and Darwinian views of evolution. J Mol Biol.

[58] Wargo AR, Kurath G (2012). Viral fitness: definitions, measurement, and current insights. Curr Opin Virol.

[59] Murtaugh M, editor. PRRS immunology: what are we missing? Annual Meeting of American Association of Swine Veterinarians. Des Moines; 2004.

[60] Murtaugh MP, Stadejek T, Abrahante JE, Lam TTY, Leung FCC (2010). The ever-expanding diversity of porcine reproductive and respiratory syndrome virus. Virus Res.

[61] Liu Y, Li J, Yang J, Zeng H, Guo L, Ren S (2018). Emergence of different recombinant porcine reproductive and respiratory syndrome viruses, China. Sci Rep.

[62] Moura C, Johnson C, Baker S, Holtkamp D, Wang C, Linhares D (2019). Assessment of immediate production impact following attenuated PRRS type 2 virus vaccination in swine breeding herds. Porcine Health Manag.

[63] Lebret A, Berton P, Normand V, Messager I, Robert N, Bouchet F (2021). PRRSV detection by qPCR in processing fluids and serum samples collected in a positive stable breeding herd following mass vaccination of sows with a modified live vaccine. Porcine Health Manag.

[64] Hsueh FC, Wang SY, Lin WH, Lin CF, Tsai CY, Huang CW (2021). Correlation of neutralizing antibodies (NAbs) between sows and piglets and evaluation of protectability associated with maternally derived NAbs in pigs against circulating porcine reproductive and respiratory syndrome virus (PRRSV) under field conditions. Vaccines (Basel)..

[65] Galvis JA, Prada JM, Corzo CA, Machado G. Modeling the transmission and vaccination strategy for porcine reproductive and respiratory syndrome virus. Transbound Emerg Dis. 2021. 10.1111/tbed.14007 33506620

[66] Turner M (2005). Mass vaccination applied to a production system.

[67] Gillespie T, Carroll A (2003). Methods of control and elimination of porcine reproductive and respiratory syndrome virus using modified live vaccine in a two-site production system. J Swine Health Prod.

[68] Murtaugh M, Yuan S, Nelson E, Faaberg K (2002). Genetic interaction between porcine reproductive and respiratory syndrome virus (PRRSV) strains in cell culture and in animals. J Swine Health Prod.

[69] Loula T. What’s new with PRRS on commercial farms? In: Allen D Leman Swine conference; 1998. p. 172–3.

[70] Ladinig A, Wilkinson J, Ashley C, Detmer SE, Lunney JK, Plastow G (2014). Variation in fetal outcome, viral load and ORF5 sequence mutations in a large scale study of phenotypic responses to late gestation exposure to type 2 porcine reproductive and respiratory syndrome virus. PLoS ONE.

[71] Rowland RR, Lawson S, Rossow K, Benfield DA (2003). Lymphoid tissue tropism of porcine reproductive and respiratory syndrome virus replication during persistent infection of pigs originally exposed to virus in utero. Vet Microbiol.

[72] Eclercy J, Renson P, Lebret A, Hirchaud E, Normand V, Andraud M (2019). A field recombinant strain derived from two type 1 porcine reproductive and respiratory syndrome virus (PRRSV-1) modified live vaccines shows increased viremia and transmission in SPF pigs. Viruses.

[73] Kvisgaard LK, Kristensen CS, Ryt-Hansen P, Pedersen K, Stadejek T, Trebbien R (2020). A recombination between two type 1 porcine reproductive and respiratory syndrome virus (PRRSV-1) vaccine strains has caused severe outbreaks in Danish pigs. Transbound Emerg Dis.

[74] Linhares DC, Cano JP, Wetzell T, Nerem J, Torremorell M, Dee SA (2012). Effect of modified-live porcine reproductive and respiratory syndrome virus (PRRSv) vaccine on the shedding of wild-type virus from an infected population of growing pigs. Vaccine.

[75] Moura CAA, Philips R, Silva GS, Ramirez A, Gauger PC, Holtkamp DJ (2021). Association of wild-type PRRSV detection patterns with mortality of MLV-vaccinated growing pig groups. Prev Vet Med.

[76] Harding JCS, Ladinig A, Novakovic P, Detmer SE, Wilkinson JM, Yang T (2017). Novel insights into host responses and reproductive pathophysiology of porcine reproductive and respiratory syndrome caused by PRRSV-2. Vet Microbiol.

[77] Ladinig A, Ashley C, Detmer SE, Wilkinson JM, Lunney JK, Plastow G (2015). Maternal and fetal predictors of fetal viral load and death in third trimester, type 2 porcine reproductive and respiratory syndrome virus infected pregnant gilts. Vet Res.

[78] Harris DLH (2000). Multi-site pig production.

[79] Jara M, Rasmussen DA, Corzo CA, Machado G (2020). Porcine reproductive and respiratory syndrome virus dissemination across pig production systems in the United States. Transbound Emerg Dis.

[80] Moura C, Holtkamp D, Linhares D, editors. Production and economic benefit of a full PRRSV MLV dose compared to a partial dose vaccination program on nursery pigs. In: 2019 Leman conference. Minneapolis; 2019.

[81] Perez A, Davies P, Goodell C, Holtkamp D, Mondaca-Fernandez E, Poljak Z (2015). Lessons learned and knowledge gaps about the epidemiology and control of porcine reproductive and respiratory syndrome virus in North America. JAVMA..

[82] Perez AM, Alba A, Goede D, McCluskey B, Morrison R (2016). Monitoring the spread of Swine enteric coronavirus diseases in the United States in the absence of a regulatory framework. Front Vet Sci.

[83] VanderWaal K, Perez A, Torremorrell M, Morrison RM, Craft M (2018). Role of animal movement and indirect contact among farms in transmission of porcine epidemic diarrhea virus. Epidemics Neth.

